# The value of replication: commentary on “Replication and methodological robustness in quantitative typology” by Becker and Guzmán Naranjo

**DOI:** 10.1515/lingty-2025-0019

**Published:** 2025-07-30

**Authors:** Matthew S. Dryer

**Affiliations:** 12292 University at Buffalo, Buffalo, NY, USA; University of Alberta, Edmonton, Canada

While the increasing number of typological studies using more sophisticated statistics is certainly progress, it is often not clear how confident we should be in accepting the results because it is often not clear whether the authors have applied the method correctly or controlled adequately for factors such as genealogy and geography. In fact, until studies have been replicated using alternative methodologies, I would argue that linguists should be wary of all typological studies using statistics until the results have been replicated. In my opinion, the highest priority in typology is not new results but replication of existing studies, using different methodologies. That is why Becker and Guzmán Naranjo’s paper (2025) (hereafter B&GN) is exactly what is needed.

When a replication of a study using a different method gets results that are similar to those of an original study, we not only have greater confidence in the results of the original study, but also greater confidence in the two methods employed in the original study and its replication. Conversely, when two methods yield somewhat different results, we need to find the source of the difference to determine if the difference reflects a weakness in one of the two methods. B&GN’s discussion of my 2018 paper (Dryer 2018) provides a perfect example of this. They observe that while both methods find the type N-A-Num-Dem to be the most common order cross-linguistically, their estimated frequency of this type is substantially higher than in my study. Restating B&GN’s explanation for the difference, the source of the difference can be described as follows. Although my method uses a large number of samples in determining the frequency of different types, a property of each sample is that it cannot include two languages that are, loosely speaking, genealogically or geographically close to each other. What this means is that if a small area includes a large number of languages genealogically or geographically close to each other that are all of the same type, no sample in my method would include more than one language from this area. What this means, in turn, is that my method does not distinguish such an area from a similar area where only one of the many languages in that area is of a given type. My estimate of the frequency of the N-A-Num-Dem type apparently misses the fact that there are many such small areas where most of the languages are of this type, whereas B&GN’s method does not suffer in this respect. In other words, we can identify the source of the difference and identify which method provides a more reliable result.

**Figure 1:**
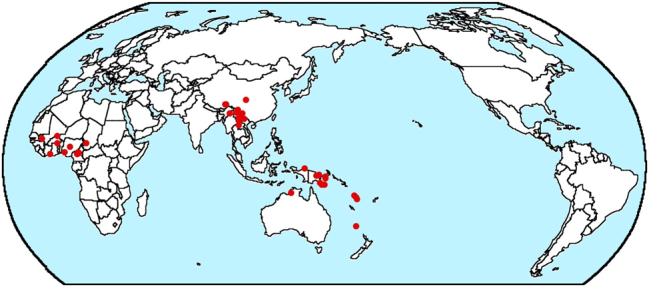
Map of Dem-Num-N-A languages © The author.

The source of a second difference between B&GN’s results and mine is less clear. My estimate of the frequency of another type, Dem-Num-N-A, is higher than theirs. B&GN provide a map (their Figure 3) which shows, they claim, that this type is restricted to a number of areas and that my method misses the areality of the distribution of this type. They observe that this type is restricted to “Western Europe, Eastern Turkey, Amazonia, Central Mexico, and potentially North America”. But their map shows that there are still a number of languages of this type outside these areas, one in northeast India, one in the Andamans, two in New Ireland, one in Hawaii, one in northern Mexico, one in Costa Rica, two near the west coast of South America (which, in my experience, tends to pattern rather differently from Amazonia), and one in Tierra Del Fuego. Furthermore, most of the other word order types in my study exhibit a similar degree of areality if not more. For example, the type that is next in frequency to Dem-Num-N-A in my study is N-A-Dem-Num (with 36 languages compared to 40 for Dem-Num-N-A). The map in Figure 1 from my database exhibits a higher degree of areality for this type in that all the instances of this type are found in three areas, west Africa, an area in southeast Asia, and the area around New Guinea (though it is not clear that the last of these should be considered a single area).1The maps in this paper were produced by my typological database.

**Figure 2:**
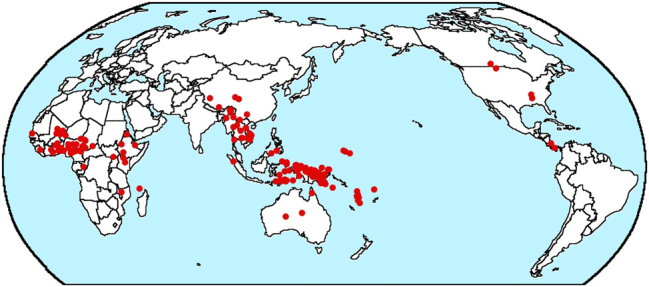
Map of N-A-Num-Dem languages © The author.

In fact, the map from my database for the most frequent type, N-A-Num-Dem, shown in Figure 2, also shows a high degree of areality, with most of the languages in Subsaharan Africa, southeast Asia (though extending well into Tibet) and the area around New Guinea. There are no instances in Eurasia outside of the area in southeast Asia just noted and only six instances in all of the Americas.

Hence it is not clear that the distribution of Dem-Num-N-A is any more areally restricted than other types and hence unclear that B&GN are correct is suggesting that the different results reflect a failure of my method to detect the areality of this type.

What I suspect is the actual source of the difference between B&GN’s results and mine is the opposite of what B&GN suggest. Rather than my method failing to detect the amount of areality of Dem-Num-N-A languages, I suspect that my method is failing to detect the relative lack of areality of Dem-Num-N-A languages. I observed above that my method fails to distinguish a case involving a large number of instances of a type that are genealogically or geographically close to each other from a single instance of a type with no other instances close by. What this means is that if many instances of a type are of the latter sort, i.e. a relatively large number of isolated instances of the type, my method will overestimate the frequency of the type. I suspect that it is the number of instances of Dem-Num-N-A languages that are outside the areas B&GN mention that is the source of the difference between B&GN’s method and mine.

The real challenge for typological studies estimating the frequency of different types is (and always has been) controlling for geography. B&GN attempt to control for geography by using the Euclidean distance between languages (or more accurately, the Euclidean distance between the centre of languages, since the locations listed in Glottolog involve points, not areas). However, as pointed out to me by Michael Cysouw (pers. comm.), being 200 km apart in New Guinea is very different from being 200 km apart in Siberia.2Cysouw et al. (2012) make a similar point, comparing Siberia to Africa, though comparing Siberia to New Guinea seems more appropriate given that language density varies considerably within Africa.
Cysouw et al. (2012) propose that we ought to be measuring distance, not in terms of Euclidean distance, but in term of a measure of distance that considers language density. In Dryer (2018), I distinguish what I call absolute distance, which is the distance in kilometres, from what I call relative distance, which controls for language density. I define the relative distance between two languages A and B as the number of languages C such that the distance from A to C and the distance from B to C are both less than the distance from A to B.

In Dryer (2018), I use both absolute distance and relative distance in controlling for geography, because Cysouw is only half-right in pointing out the problem with absolute distance. The reason that he is only half-right is that it is also the case that being ten languages apart is very different in New Guinea than it is in Siberia.3In one of the locations in Papua New Guinea where I have done fieldwork, it is common for women to marry into villages where the language is more than ten languages away from their native languages (using my definition of relative distance). I suspect that this is more unusual in Siberia. This is relevant if one of the sources of contact-induced change is women marrying into village where the dominant language is different from their native language and the women are passing features of their native language on to their children. So neither absolute distance nor relative distance alone is sufficient for controlling for geography. B&GN control for geography only using absolute distance. A question arises, therefore, whether they have adequately controlled for geography. What is needed is a replication of B&GN’s replication using both absolute and relative distance.

**Figure 3:**
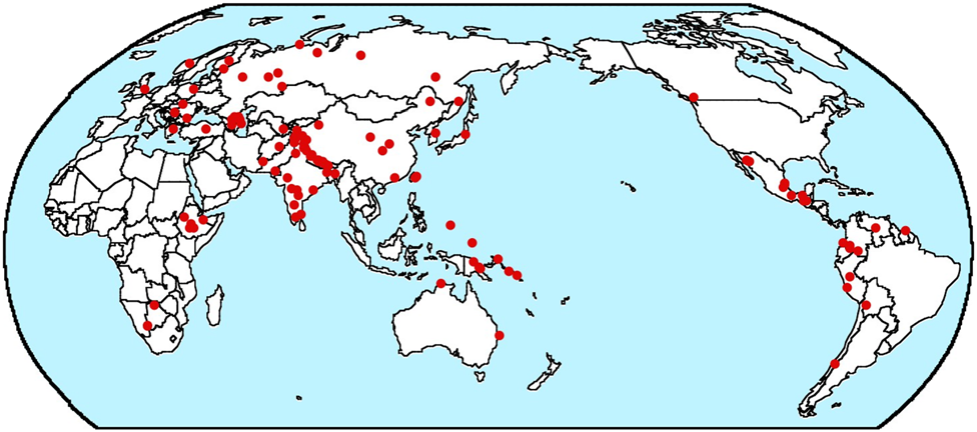
Map of Dem-Num-A-N languages © The author.

But I have another worry about whether both B&GN and my 2018 paper fail to adequately control for geography. In Dryer (1989), I argue that there are very large linguistic areas encompassing most of entire continents. And the distribution of N-A- Num-Dem order shown on the map in Figure 2 shows most of the cases falling in three large areas. But what if history had been different and it had been Dem-Num-A-N that was so common in Africa rather than its mirror opposite? Would we then be trying to explain why Dem-Num-A-N order is much more common than N-A-Num-Dem order? Or consider the distribution of Dem-Num-A-N order in the map in Figure 3.

The map in Figure 3 shows a different sort of areality from that in Figure 2 for N-A-Num-Dem languages. In the latter case, there are three areas where the order is common and relatively few instances elsewhere. But in the case of Dem-Num-A-N in Figure 3, there is a single large area where the order is common and a sprinkling of other instances scattered across the rest of the world. In fact over 60 % of the Dem-Num-A-N languages in my study (71 out of 113) are spoken in Eurasia (which for my purposes, excludes languages of Indonesia, the Philippines, and Taiwan). What if history had been different and the languages in this large area had been N-A-Num-Dem? Would we then be trying to explain why Dem-Num-A-N order is relatively rare compared to N-A-Num-Dem?

In describing the Gaussian process they use for controlling for geography, B&GN say “the strength of influence quickly drops to zero for observations which are further apart”. It is unclear how quickly B&GN mean when they say the influence due to contact quickly drops to zero. But the existence of large linguistic areas suggests that some areal phenomena include languages that are more distant from each other geographically. Of course, the spread of features due to contact occurs at the local level so that large linguistic areas are in one sense just a series of instances of contact influence among languages that are close to each other. However, the existence of large linguistic areas suggests that sometimes the spread of a feature due to contact has a kind of snowball effect in that sometimes the probability of contact-influenced change increases after a change has started to spread. It is unclear whether B&GN’s model captures this.
